# Influence of *Albizia lebbeck* Saponin and Its Fractions on *In Vitro* Gas Production Kinetics, Rumen Methanogenesis, and Rumen Fermentation Characteristics

**DOI:** 10.1155/2014/498218

**Published:** 2014-03-04

**Authors:** Sunil Kumar Sirohi, Navneet Goel, Nasib Singh

**Affiliations:** ^1^Dairy Cattle Nutrition Division, National Dairy Research Institute, Karnal, Haryana 132001, India; ^2^Lovely Professional University, Jalandhar, Punjab 144411, India

## Abstract

The present study was undertaken to investigate the effect of crude seed powder (CSP) and gross saponins extract (GSE) of seeds of *Albizia lebbeck* on antimicrobial activity by taking two Gram-positive (*Staphylococcus aureus* and *Bacillus cereus*), two Gram-negative (*Escherichia coli* and *Salmonella* Typhi) bacteria, and two fungi species (*Aspergillus niger* and *Candida butyric*) were taken at 25, 50, 100, 250, and 500 µg levels using agar well diffusion method. Zone of inhibition was increased with increasing of concentration of CSP and saponins which indicates that Gram-negative bacteria (*E. coli*), Gram-positive bacteria (*B. cereus*), and *A. niger* were significantly susceptible to inhibition. Another experiment was conducted to study the effect of GSE and saponins fraction A and B of *A. lebbeck* supplementation at 6% on DM basis on methane production and other rumen fermentation parameters using *in vitro* gas production test, by taking three different type diets, that is, high fiber diet (D1, 60R : 40C), medium fiber diet (D2, 50R : 50C), and low fiber diet (D3, 40R : 60C). Significant (*P* ≤ 0.05) increase was seen in IVDMD, methane production; however ammonia nitrogen concentration decreased as compared to control. The methane production was reduced in a range between 12 and 49% by saponin supplemented diets except in case of GSE in D2. Sap A showed the highest methane reduction per 200 mg of truly digested substrate (TDS) than other treatment groups. Results in relation with quantification of methanogens and protozoa by qPCR indicated the decreasing trend with saponins of *A. lebbek* in comparison with control except total methanogen quantified using *mcr-A based* primer.

## 1. Introduction

Methane production during anaerobic fermentation of nutrients in the rumen is an essential metabolic but nutritionally wasteful process which represents 2 to 15% of gross energy loss [1, 2]. Livestock contributes 12–18% to the global anthropogenic greenhouse gas emissions depending upon emission attributes [3, 4] and accounts for about 37% of the total anthropogenic methane [3]. Therefore, reducing methane production is an important goal of ruminant nutritionists not only for reducing greenhouse gases and global warming but also for improving the efficiency of animal production. Recently, researchers have emphasized on the reduction of methane emission by using ionophores, organic acids, fatty acids, plant extracts, and halogenated methane analogues could be used to decrease ruminal methane production [5–12]. Plant secondary metabolites, that is, saponins, tannins, and essential oils have also been widely studied for their beneficial effect on ruminants and several workers reported that saponins and plants rich in saponins decreased the methane production in the rumen [11, 13–15]. Hence, the present investigation was carried out to assess the effect of gross as well as pure saponins fraction of *Albizia lebbeck* on antimicrobial potential on selected bacterial and fungal pathogens, rumen fermentation characteristic, and antimethanogenesis.

## 2. Materials and Methods

### 2.1. Extraction, Isolation, and Estimation of Saponins

#### 2.1.1. Plant Materials

Seeds of* Albizia lebbeck* were collected from the campus of National Dairy Research Institute, Karnal, India. Seeds were washed with distilled water, dried at 50°C in hot air oven for 24 h, then ground in hammer mill to pass through 1 mm sieve. The powder was stored in an air-tight container until use.

#### 2.1.2. Extraction and Isolation of Saponins

Seed powder was defatted in petroleum ether (boiling range 40–60°C) refluxing for 6 h in a soxhlet's apparatus at 45°C. The contents were filtered and 25 g fat-free sample was diluted with absolute methanol taken in 1 : 10 ratio. The contents were shaken at 25°C and 120 rpm for 24 h followed by centrifuge for 20 min at 3500 rpm for 20 min. Methanol extract was filtered through Whatman filter paper Number 1 and dried under rotary evaporator. Dried methanolic plant extract was dissolved in distilled water (10 mL), transferred in to a separating funnel, and extracted with equal volume of *n*-butanol (3 times). Again, solvent, *n*-butanol was further evaporated at 45°C. Dried saponins content was dissolved 5–10 mL of distilled water freeze dried resulting in a yellowish amorphous powder designated gross saponins extract (GSE).

Gross saponins extract (GSE) was extracted by previously described method [16]. GSE was fractionated by applying on silica gel (mesh range 60–120) column chromatography and eluted successfully with CHCl_3_-MeOH (9 : 1) to yield fraction A; subsequent elution with CHCl_3_-MeOH-H_2_O (40 : 10 : 1) to give the fraction B.

#### 2.1.3. Estimation of Total Saponins

Total saponins contents of *A. lebbeck* seeds were estimated by colorimetric methods [17]. Gross saponins extract (10 mg) was dissolved in 5 mL of 80% aqueous methanol and 50 *μ*L of this solution was taken in different test tubes to which 0.25 mL of vanillin reagent (8%, w/v in 99.9% ethanol) was added. Test tubes were placed in ice-cold water bath and 2.5 mL of 72% (v/v) sulphuric acid was added slowly on the inner side of the wall. After mixing the content in each tube, then left as such for 3 min, then warmed the tubes at 60°C for 10 min using water bath and cooled in ice-cold water bath. Absorbance was measured at 544 nm using spectrophotometer against the reagent blank and standard curve was prepared. Quillaja saponin (Sigma-Aldrich) was used as a reference standard [18] and the concentration of total saponins was expressed as Quillaja saponin equivalents (QS *μ*g/mg extract).

### 2.2. Antimicrobial Study (Experiment 1)

#### 2.2.1. Microbial Cultures

Two Gram-positive and two Gram-negative bacteria and two fungi species were employed to determine the antimicrobial action of *A. lebbeck *seed powder extracted saponins. All microbial cultures were taken from National Collection Centre, Dairy Microbiology Division, National Dairy Research Institute, Karnal, India, and Microbial Type Culture Collection, IMTECH, Chandigarh, India.

#### 2.2.2. Antimicrobial Activity Assay

Antibacterial and antifungal activities of the crude seed powder (CSP) and gross saponins extract (GSE) were tested using agar well diffusion method as described previously [19]. Nutrient agar/BHI agar was used for the determination of antibacterial and antifungal activities. The samples were tested at 25, 50, 100, 250, and 500 *μ*g concentrations of treatments. On agar plates test bacterial cultures (10^8^ cfu/mL) were spread with sterilized loop and incubated at 37°C for 3 h. Wells of 8 mm size were punched on plates using sterile borer. Different dilutions of test samples were then added to the wells in approximately 100 *μ*L volume. Following incubation for 24–48 h at 37°C, the sensitivity of the bacterial species to the saponins was determined by measuring the diameter of the zone of inhibition around the well. Each sample was assayed in triplicate. Chloramphenicol (30 *μ*g/well) and Kanamycin sulphate (50 *μ*g/well) were used as possible control for Gram-positive bacteria and Gram-negative bacteria, respectively.

Suspensions of fungal spores were prepared from 5–7 days old cultures that grew at 28°C on a SDA plates which were prepared by pour plating using fungal spores of *A. niger* as inoculums. An aliquot to this inoculum was introduced to molten SDA and poured into petri dishes. *C. butyri* agar plates were also prepared by pour plating. Wells of 8 diameters were punched and test samples were introduced at different concentration as described in the previous section. Plates were incubated for 24–48 h at 28°C for *A. niger* and at 35°C for *C. butyri*. The antifungal activity was evaluated by measuring zones of inhibition of fungal growth surrounding the wells. Each sample was assayed in triplicate and Nystatin (50 *μ*g/well) was used as positive control.

### 2.3. *In Vitro* Rumen Fermentation (Experiment 2)

#### 2.3.1. Diets

The substrates used in incubation were prepared by taking different roughage and concentrate ratio, that is, high fiber diet (D_1_, 60R:40C), medium fiber diet (D_2_, 50R:50C), and low fiber diet (D_3_, 40R:60C) and milled to pass through 1 mm sieve and used as substrate. The roughage part composed of wheat straw and the concentrate part composed of maize (33%), GNC (21%), mustard cake (12%), wheat bran (20%), deoiled rice bran (11%), mineral mixture (2%), and common salt (1%), respectively.

#### 2.3.2. Experimental Design and Technique

All the treatments, that is, 6% (DM basis) of gross saponins extract (GSE) and saponin fractions A and B were arranged in factorial randomized block design (RBD) with three replicates. Sets were also incubated devoid of substrate with and without supplementation which served as blanks for particular treatment and values were corrected for different parameters with blanks. The experiment was conducted in 100 mL calibrated glass syringes containing 200 ± 5 mg of substrates with 6% GSE and 6% of saponins fraction A and B, respectively, and then 30 mL reduced buffer medium [20] was anaerobically added to each syringe. Syringes were incubated at 39°C for 48 h in temperature-controlled water bath cum shaker. The rumen liquor was from a fistulated adult male buffalo (*Bubalus bubalis*) maintained on a standard diet (roughage: concentrate; 60 : 40) 1 h before morning feed. Strained rumen liquor was collected in sterile, prewarmed and pre-CO_2_ flushed insulated thermos flask and brought to the laboratory immediately. All animal procedures were performed in accordance with the guidelines of Institutional Animal Ethics Committee of National Dairy Research Institute, Karnal (India).

#### 2.3.3. *In Vitro* Total Gas (TG) Production and Methane Estimation

After 48 h incubation, total gas (TG) was estimated by the extent of displacement of piston of glass syringes. TG produced due to fermentation of substrate was corrected by subtracting TG produced in blank syringe (containing inoculum and buffer but not the substrate) from total gas produced in the syringe containing substrate, inoculum, and buffer. Methane concentration in representative gas samples was estimated by using gas chromatograph (Nucon-5765, India) equipped with flame ionization detector (FID) and stainless steel column packed with Porapak-Q (length 6′; o.d.1/8” i. d. 2 mm; mesh range 80–100). The gas flow rates for nitrogen, hydrogen, and air were 30, 30, and 300 mL/min, respectively. Temperature of injector oven, column oven, and detector were 40, 50, and 50°C, respectively. CH_4_ in samples were calculated by external calibration, using a certified gas standard mixture of 50% CH_4_ and 50% CO_2_ (Spantech, England).

#### 2.3.4. Measurements of Digestibility and Fermentation Parameters

The true DM degradability of feed sample of each syringe containing residues after incubation was estimated as per method [21]. The proximate analysis (organic matter, crude protein, ether extract, and total Ash) of substrate was carried out as per the method [22]. The cell wall constituents of substrates were determined according to described method [23]. For determination of NH_3_-N, 5 mL of supernatant was taken in tube mixed with 12 mL 1N NaOH and steam passed using KEL PLUS-N analyzer (Pelican, India) and the NH_3_ evolved was collected in conical flask containing boric acid solution having mixed indicator and titrated against N/100 H_2_SO_4_.

For the estimation of individual volatile fatty acids, 4 mL of 25% metaphosphoric acid was added to 1 mL of incubation sample; the mixture was mixed uniformly and left as such for 3-4 h at ambient temperature [24]. Thereafter, samples were centrifuged at 3000 rpm for 10 min and clear supernatant was stored at −20°C until analyzed. The volatile fatty acids were analyzed by using gas liquid chromatography (Nucon-5765, New Delhi, India) after some modification of the previously described method [6].

#### 2.3.5. Estimation of Partition Factor (PF) and Microbial Biomass Production (MBM)

The PF is calculated as the ratio of substrate truly degraded *in vitro *(mg) to the volume of gas (mL) produced by it. Substrate provides important information about partitioning of fermentation products. The MBM yield was calculated by using the degradability of substrate and gas volume and stoichiometrical factor as suggested [25]:

(1) Microbial  mass=Substrate  truly  degraded −(gas  volume   ×stoichiometrical  factor),

where the stoichiometrical factor used was 2.25.

#### 2.3.6. Quantitative Real-Time PCR (qRT-PCR) Quantification of Methanogens

Content of the glass syringes containing D_2_ with treatment was shaken thoroughly and one mL samples was withdrawn at 48 h of the experiment. Total genomic DNA was extracted using genomic DNA extraction kit (Fermentas, USA) as per manufacturer's instructions. DNA concentrations were measured in NanoQuant instrument (Tecan, USA). In order to minimize the variations, DNA was extracted from all three samples. qRT-PCR was performed to quantify total rumen methanogens, methanomicrobiales, and protozoa. Assays were performed in MJ Mini Mini Opticon Real-Time PCR System (Bio-Rad, USA) using SYBR Green Jump Start Taq Readymix (Sigma, USA). The primer pairs used for different microbial groups are described in Table 5. Samples were assayed in 25 *μ*L reaction mixture containing 5 mM MgCl_2_, SYBR Green master mix, 50 ng of template DNA, and 0.5 *μ*M of each primer. All assays were performed in triplicate.

#### 2.3.7. Gas Production Kinetics

The total gas production kinetics and cumulative methane gas production were carried out in D_2_ with different treatment combinations and incubated as per the procedure mentioned above for different intervals, that is, 0, 1, 2, 3, 6, 9, 12, 24, 36, 48, 60, 72, and 96 h. Kinetics of gas production was calculated using a nonlinear model [26]. The NLIN procedure of Sigma stat 3.11 was used to fit the following model: *p* = *b*[1^−*e*−*c*(*t*)^], where *p* is the gas production rate at time *t*, *b* is the potential gas production (mL), and *c* is gas production rate constant (mL/h) of *b* and *t* is the time of incubation (h). The potential gas production and rate of gas production were calculated by fitting the modified equation [26].

### 2.4. Statistical Analysis

Experimental data of different parameters were analyzed in randomized block design with three replicates for analysis of variance [27]. The effects of gross saponins and different saponins fraction compared with controls were tested using the factorial arrangement in randomized block design in OPSTAT (http://14.139.232.166/opstat/index.asp) statistical software developed by Chaudhry Charan Singh, Haryana Agriculture University, Hissar, Haryana, India [28].

## 3. Results and Discussion

### 3.1. Antimicrobial Activity

The crude seed powder (CSP) and gross saponins extract (GSE) of *A. lebbeck* seed exhibited significant antimicrobial activities against bacterial and fungal cultures (Table 1). The extent of inhibition was greater in the case of pure saponin fraction than crude saponins fractions.

The results of present experiments indicated that the zones of inhibition for Gram-negative and Gram-positive bacteria were increased with increasing of concentration of treatments, that is, CSP and GSE. Gram-negative bacteria *E. coli* and Gram-positive bacteria *B. cereus* were more susceptible to inhibition than other tested bacteria to CSP and GSE. *E. coli* showed the maximum zone of inhibition (12.8 mm, 7.2 mm), while, *B. cereus* showed the maximum (13.3 mm, 7.8 mm) at 500 *μ*g levels of GSE and CSP, respectively. Gram-positive bacteria *B. cereus* was more susceptible to inhibition in comparison with Gram-negative bacteria *E. coli*. GSP and GSE were also used for the evaluation of their antifungal activity against *Candida butyri *and* Aspergillus niger*. *A. niger* was significantly susceptible to inhibition by saponins fraction and showed the highest inhibition 11.8 and 6.7 mm at 500 *μ*g level of GSE and CSP, respectively. *C. butyri* was least inhibited by saponin fractions and showed the highest inhibition (3.2 mm) at 500 *μ*g level of GSE and did not showed any activity at low level of GSE as well as CSP. The results of present study indicated that gross saponins fraction of *A. lebbeck* showed the inhibitory action against Garm-positive bacteria but not show significant inhibition against Gram-negative bacteria and fungi. This is not surprising because the Gram-negative bacteria and fungi have been shown to be more resistant to antibiotics [29, 30]. This may possibly be the presence of high lipid content in the cell walls of Gram-negative bacteria and saponins may not be able to penetrate the cell membrane of the microorganism [31, 32]. The finding of present study was consistent with previous published reports that specifically showed that saponins could have antimicrobial properties [33–35].

### 3.2. *In Vitro* Dry Matter Digestibility (IVDMD) and Rumen Fermentation Parameters

The ingredient and chemical compositions of diets containing different roughage and concentration ratio were presented in Table 2. *In vitro* results of incubating three diets during 48 h with GSE, saponin fraction A and B on digestibility, rumen fermentation, methanogenesis, and so forth were presented in Tables 3 and 4, respectively.

#### 3.2.1. IVDMD, Partition Factor and Microbial Biomass

In the current experiment, results indicated that IVDMD values were increased as compared to control and the differences among treatments values were significant (*P* ≤ 0.05) except GSE inclusion in D_2_ and D_3_, where slight reduction in IVDMD was observed. In case of D_1_ and D_3_, IVDMD was increased with 15.33% and 2.05% by supplementation of saponin fractions A, while in D_2_ highest 7.38% increase of IVDMD was noticed on inclusion of saponin fraction B of *A. lebbeck* seeds. Digestibility increase as a result of the presence of saponins was similar to the previous studies [36], in which the apparent dry matter digestibility increased on supplementation of surfactant saponins at the levels of 5–20 *μ*L/g dry matter. Another study reported that the IVDMD was not affected significantly (*P* < 0.05) on the addition of pure saponins [37].

In present study the partition factor (PF) values and microbial biomass (mg) production decreased with all saponins supplementation in D_2_ and D_3_; however, in D_1_, 21.99% and 55.92% increases in PF and MBM were observed during supplementation of saponins fraction A (Table 3). This finding was in accordance with the finding of Goel et al. [38]. They reported that the MBM and PF increased on inclusion with extracted saponins from *Achyranthes aspera, Tribulus terrestris,* and *Albizia lebbeck* at 3, 6, and 9% levels on DM basis.

#### 3.2.2. Methane Production

Results of present study indicated that methane production was decreased in saponins extracted from *A. lebbeck* seed supplementation and in different diets; methane production (mL/gDM) was reduced approximately in the range 12 to 49%, except GSE with D_2_. Results of current study showed that the methane production was reduced up to 49% which was in accordance with results of several experiments conducted by different workers [14, 39–41]. In another study, Holtshausen et al. [42] reported in study with *Yucca schidigera plant* extract containing 6% saponins which showed 8.5% methane reduction at the level of 0.38 g/liter. Similarly, Feng et al. [43] showed that gross saponin of *Tribulus terrestris* at 0.3, 0.6, and 0.9 g/liter levels significantly (*P* < 0.05) reduced methane concentration by 23.43, 24.93, and 25.30%, respectively, by *in vitro* gas production technique.

Results of the present experiment showed that the reduction of methane production per 200 mg of truly digested substrate (TDS) was highest in saponin fraction A with all diets, when compared with control (Table 3). These results were in agreement with the earlier finding. Castro-Montoya et al. [44] showed that addition of *Quillaja* saponin reduced the methane by 4.1 mL/100 mg substrate at 1.25 mg of saponin/liter under *in vitro* studies.

In the rumen, the methane production also depends upon the association between methanogens, protozoa, and rate of methane production per methanogenic cell [45]. Patra and Saxena [15] suggested that saponin may decrease the protozoal numbers which leads to reducing the availability of hydrogen ions for methane production by methanogens. Furthermore, it has been shown that the saponins reduced methane production via diminished activity of methane producing gene without changing the total methanogen population [41].

#### 3.2.3. Short Chain Fatty Acid (SCFA) and Ammonia Nitrogen

The individual volatile fatty acids (IVFAs) concentration varied among the treatments (Table 4). Acetate concentration increased on inclusion of all treatments in case of D_2_ and D_3_ and highest increased (15.66%) was seen in saponin fraction B supplementation in D_2_. While in D_1_, acetate production was decreased in all treatments.

Results of current experiment indicated that the concentration of propionate slightly was affected by *A. lebbeck* seed saponins. In D_2_, it was increased in all treatments and increased most with saponins fraction B (36.23%) in comparison to control (Table 4). Nonsignificant change in butyrate concentrations were observed in present study. Among all three diets, only D_2_ showed the slight decrease in A/P ratio; however, in case of D_1_ and D_2_ it was increased and highest increase (27.71%) was seen in D_3_ on supplementation with saponin fraction B. The results of present study were in accordance with several other studies [13, 46]. In another study, Istiqomah et al. [47] observed that the acetate to propionate ratio decreased at 5, 10, and 15% saponin levels. Similarly, Feng et al. [43] also observed that the saponin level at 0.9 g/L decreased the acetic acid and at 0.6 and 0.9 g/L increased the propionic acid concentration significantly when compared to the control.

The ammonia nitrogen (mg/100 mL) was decreased due to the *A. lebbeck* seed saponins in all three diets, and the maximum decrease (38.71%) was found in D_3_ on supplementation with saponin fraction B (Table 4). The results of current study indicated that the ammonia nitrogen was marginally affected and slightly decreased as compared to control diet without supplementation of saponins. Results were consistent with earlier reports [37, 43, 48]. Bharathidhasan et al. [37] observed that nonsignificant reduction in ammonia nitrogen on inclusion with purified saponins at the levels of 0, 1.55, 3.10, 4.65 and 6.20 mg/30 mL rumen inoculums.

#### 3.2.4. Gas Kinetics of Total Gas and Methane Production

**Figure 1:**
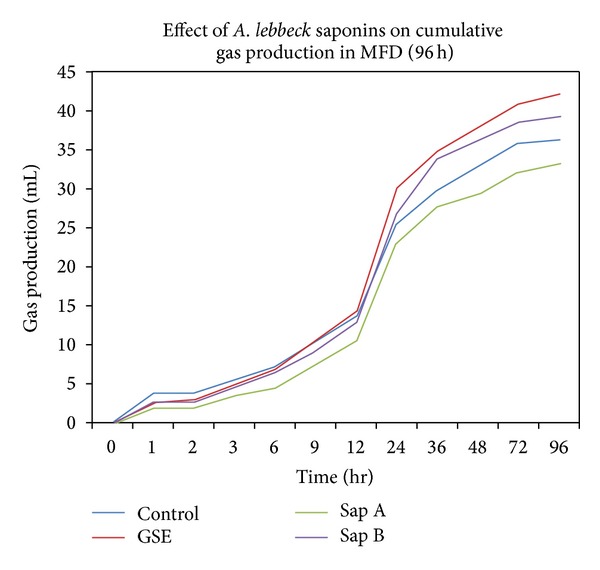
Effects of *A. lebbeck* saponins fractions on total gas potential (96 h) using D_2_ as a substrate.

Results related to gas kinetics in D_2_ diet are presented in Table 5. Gas kinetics results showed that potential gas production (b) was increased on supplementation with GSE (18.13%) and saponin fraction B (12.17%), while slight decrease was noticed on saponin fraction A inclusion in comparison to control (Figure 1). On the other hand, the gas production rate (c) was decreased in all treatments and highest decrease (15.81%) was noticed on inclusion of saponin fraction A in diets.

**Figure 2:**
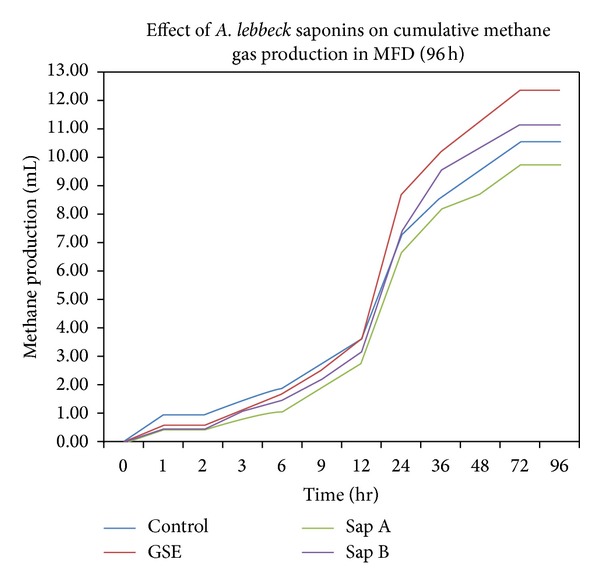
Effects of *A. lebbeck* saponins fractions on methane gas potential (96 h) using D_2_ as a substrate.

In current study, the results of cumulative methane gas production (mL/gDM) were presented in Table 6 and trends were similar to gas kinetics. Highest (20.35%) increase in methane gas potential (b) and highest reduction (13.54%) in methane production rate were observed on supplementation with GSE and saponin fraction A, respectively (Figure 2).

#### 3.2.5. Quantification of Methanogens and Protozoal Population

In present study, results of quantification of methanogens and protozoal population are presented in Table 7. Results indicated that all treatments show the antiprotozoal effect and maximum reduction in protozoa population (35.1%) was seen in saponin fraction A, when compared to control diet. The results of present experiment were consistent with earlier studies [14, 49, 50]. It is believed that saponins form complexes with cholesterol present in the cell membrane and result in the cell lysis, which in turn decreases the hydrogen ion transfer and ultimately reduces the methane production [51].

## 4. Conclusions

In present study it is concluded that saponins fraction A of *Albizia lebbeck* has antimethanogenic potential and has an ability to modulate the rumen fermentation parameters. However, a systematic evaluation is needed to confirm the active structural components of saponin fraction A, and their interaction with the microbial community and the diet, and to clarify the mechanism by which saponin fraction A or their metabolites exert effects on the rumen microbes.

## Figures and Tables

**Table 1:** *In  vitro* antibacterial and antifungal activities (zone of inhibition in mm) of *A.  lebback* treatments using agar well diffusion method.

Tested microorganisms			Zone of inhibition (CSP)					Zone of inhibition (GSE)					Positive control
Types	Names	Specimen number	Concentration (µg)										
			500	250	100	50	25	500	250	100	50	25	
Gram-negative bacteria	*Escherichia coli *	NCDC 135	7.2	4.8	3.3	1.3	nz	12.8	8.9	7.3	4.0	1.9	17.7^a^
	*Salmonella Typhi *	NCDC 113	3.9	2.6	1.1	nz	nz	9.2	5.9	5.7	2.8	nz	14.7^a^
Gram-positive bacteria	*Staphylococcus aureus *	MTCC 1144	3.4	2.1	0.9	nz	nz	7.4	6.2	5.0	2.3	nz	9.3^b^
	*Bacillus cereus *	NCDC 240	7.8	4.4	2.3	1.8	nz	13.3	10.1	6.3	3.3	2.0	16.7^b^
Fungi	*Candida butyri *	NCDC 280	0.8	nz	nz	nz	nz	3.2	1.2	nz	nz	nz	6.0^c^
	*Aspergillus niger *	NCDC 315	6.7	4.3	1.0	nz	nz	11.8	8.5	3.0	1.4	nz	13.2^c^

Sterile broth medium as negative control.

^a^Kanamycin sulphate (50 µg/well).

^b^Chloramphenicol (30 µg/well).

^c^Nystatin (50 µg/well).

Nz: No zone.

**Table 2:** Chemical composition of diets.

Diets	Chemical constituents of diets (g/kg on DM basis)		
	(D_1_) (60*R* : 40*C*)	(D_2_) (50*R* : 50*C*)	(D_3_) (40*R* : 60*C*)
OM	867.6	878.4	875.6
CP	108.6	125.3	142.7
EE	23.4	30.4	34.8
NDF	623.1	604.5	538.7
ADF	372.0	329.5	298.7
HC	251.1	275.0	240.0
TA	132.4	121.6	124.4

D_1_: high fiber diet, D_2_: medium fiber diet, D_3_: low fiber diet, OM: organic matter, CP: crude protein, EE: ether extract, NDF: neutral detergent fiber, ADF: acid detergent fiber, HC: hemicelluloses, TA: total Ash.

**Table 3:** Effects of *A.  lebbeck* saponins fractions on IVDMD, PF, MBM, and methane production.

Parameters	Diets														
	D_1_ (60*R* : 40*C*)				D_2_ (50*R* : 50*C*)				D_3_ (40*R* : 60*C*)				SEM		
	Control	GSE	Sap A	Sap B	Control	GSE	Sap A	Sap B	Control	GSE	Sap A	Sap B	Diet	Treatment	D ∗ T
IVDMD%	54.85 ± 0.13	61.84 ± 0.84	63.26 ± 0.18	54.62 ± 0.71	70.07 ± 0.67	68.80 ± 0.93	74.63 ± 0.79	75.24 ± 0.25	67.86 ± 0.15	63.11 ± 0.34	69.25 ± 1.24	64.25 ± 0.06	0.645	0.744	1.289
PF	3.41 ± 0.03	3.91 ± 0.08	4.16 ± 0.05	3.34 ± 0.02	3.40 ± 0.03	2.62 ± 0.06	3.04 ± 0.07	3.24 ± 0.06	3.38 ± 0.19	3.10 ± 0.07	3.52 ± 0.04	3.17 ± 0.06	0.054	0.062	0.107
MBM (mg)	37.99 ± 0.56	53.87 ± 2.06	59.83 ± 0.20	36.58 ± 0.77	47.50 ± 1.11	19.46 ± 3.16	38.63 ± 2.80	45.85 ± 1.76	46.62 ± 1.89	35.49 ± 2.05	51.83 ± 1.94	37.95 ± 2.14	1.763	2.036	3.526
CH_4_ (mL/gDM)	54.85 ± 2.17	43.74 ± 2.26	29.10 ± 1.67	34.66 ± 0.91	57.94 ± 1.96	76.59 ± 1.18	51.25 ± 0.74	50.97 ± 0.61	61.00 ± 0.36	45.37 ± 0.90	32.31 ± 2.22	31.11 ± 0.75	2.875	3.319	5.750
CH_4_ (mM/gDM)	2.20 ± 0.09	1.75 ± 0.09	1.17 ± 0.07	1.39 ± 0.04	2.32 ± 0.08	3.07 ± 0.05	2.05 ± 0.03	2.04 ± 0.02	2.44 ± 0.014	1.82 ± 0.04	1.29 ± 0.09	1.25 ± 0.03	0.115	0.133	0.230
M/TDS	0.49 ± 0.01	0.34 ± 0.02	0.22 ± 0.00	0.31 ± 0.01	0.41 ± 0.02	0.55 ± 0.01	0.34 ± 0.01	0.33 ± 0.00	0.28 ± 0.03	0.35 ± 0.01	0.23 ± 0.02	0.24 ± 0.01	0.021	0.024	0.041

D_1_: high fiber diet, D_2_: medium fiber diet, D_3_: low fiber diet, GSE: gross saponins extract, IVDMD: *in  vitro* dry matter digestibility, PF: partition factor, MBM: microbial biomass, CH_4_: methane, M/TDS: methane (mL) per 200 mg true digestible substrate; SEM: standard error of means.

**Table 4:** Effects of *A.  lebbeck* saponins fractions on volatile fatty acid and ammonia nitrogen.

Parameters	Diets														
	D_1 _(60*R* : 40*C*)				D_2_(50*R* : 50*C*)				D_3_(40*R* : 60*C*)				SEM		
	Control	GSE	Sap A	Sap B	Control	GSE	Sap A	Sap B	Control	GSE	Sap A	Sap B	Diet	Treatment	D ∗ T
Acetate (mM/100 mL)	9.24 ± 0.12	8.13 ± 0.10	8.40 ± 0.16	7.23 ± 0.30	9.19 ± 0.21	9.28 ± 0.10	9.41 ± 0.09	10.62 ± 0.03	8.02 ± 0.13	8.48 ± 0.27	8.40 ± 0.54	8.47 ± 0.32	0.165	0.191	0.330
Propionate (mM/100 mL)	2.53 ± 0.10	2.16 ± 0.08	2.25 ± 0.11	1.93 ± 0.16	2.65 ± 0.11	2.70 ± 0.14	3.00 ± 0.06	3.61 ± 0.04	2.29 ± 0.06	2.19 ± 0.14	2.48 ± 0.28	1.91 ± 0.08	0.064	0.077	0.133
Butyrate (mM/100 mL)	1.09 ± 0.06	0.89 ± 0.06	0.99 ± 0.02	0.89 ± 0.01	1.02 ± 0.04	0.94 ± 0.07	1.01 ± 0.02	1.18 ± 0.01	1.05 ± 0.04	1.18 ± 0.016	0.92 ± 0.214	0.97 ± 0.05	0.046	0.053	0.092
C2 : C3	3.65 ± 0.12	3.76 ± 0.10	3.74 ± 0.16	3.77 ± 0.30	3.48 ± 0.21	3.44 ± 0.10	3.14 ± 0.09	2.94 ± 0.03	3.50 ± 0.13	3.90 ± 0.27	3.51 ± 0.54	4.47 ± 0.32	0.119	0.137	0.238
NH_3_-N (mg/100 mL)	19.23 ± 0.09	18.48 ± 0.16	16.43 ± 0.09	15.49 ± 0.09	17.36 ± 0.16	15.87 ± 0.49	12.13 ± 0.09	10.64 ± 0.16	19.79 ± 0.09	19.13 ± 0.09	16.43 ± 0.09	16.05 ± 0.09	0.092	0.106	0.183

D_1_: high fiber diet, D_2_: medium fiber diet, D_3_: low fiber diet, GSE: gross saponins extract, C2 : C3: acetate to propionate ratio, NH_3_-N: ammonia nitrogen, SEM: standard error of means.

**Table 5:** Effects of *A.  lebbeck* saponins fractions on total gas potential (96 h) using D_2_ as a substrate.

Equation: *A* = *b* ^*X*^(1 − exp⁡ (−*c* ^*X*^ *x*))			
	*b*	*c*	*R* ^2^
Control	186.35 ± 5.07	0.215 ± 0.015	0.993
GSE	220.14 ± 8.10	0.195 ± 0.015	0.989
Sap A	175.69 ± 8.17	0.181 ± 0.020	0.986
Sap B	209.03 ± 8.44	0.191 ± 0.195	0.988

GSE: gross saponins extract, *b*: potential gas production (mL); *c*: gas production rate constant (mL/h), *R* ^2^: regression coefficient.

**Table 6:** Effects of *A.  lebbeck* saponins fractions on methane gas potential (96 h) using D_2_ as a substrate.

Equation: *A* = *b* ^*X*^(1 − exp⁡ (−*c* ^*X*^ *x*))			
	*b*	*c*	*R* ^2^
Control	55.865 ± 1.82	0.192 ± 0.015	0.992
GSE	67.24 ± 3.61	0.174 ± 0.02	0.982
Sap A	53.54 ± 3.28	0.166 ± 0.02	0.979
Sap B	61.14 ± 3.50	0.169 ± 0.02	0.981

GSE: gross saponins extract, *b*: potential methane gas production (mL); *c*: methane production rate constant (mL/h), *R* ^2^: regression coefficient.

**Table 7:** Real-time PCR quantification of changes in rumen microbial population on supplementation of *A.  lebbeck *(saponins fractions).

Microbial groups	Control diet (D_2_)	D_2_ + GSE	D2 + Sap A	D_2_ + Sap B	SEM	CD
MMB	1.00	0.318	0.484	1.581	0.091	0.321
Protozoa	1.00	0.940	0.649	0.985	0.070	0.247
mcrA	1.00	0.584	3.198	3.392	0.881	N.S.

MMB: methanomicrobiales; mcr A: total methanogens quantified using mcr A gene; in control diet population of all microbes considered as 1.00 then relative abundance is calculated with treatment.
